# Interpretation of Entropy Algorithms in the Context of Biomedical Signal Analysis and Their Application to EEG Analysis in Epilepsy

**DOI:** 10.3390/e21090840

**Published:** 2019-08-27

**Authors:** Lampros Chrysovalantis Amarantidis, Daniel Abásolo

**Affiliations:** 1 Centre for Biomedical Engineering, Department of Mechanical Engineering Sciences, Faculty of Engineering and Physical Sciences, University of Surrey, Guildford GU2 7XH, UK; 2 Ericsson, Thames Tower, Station Road, Reading RG1 1LX, UK

**Keywords:** permutation entropy, modified permutation entropy, sample entropy, quadratic entropy, fuzzy entropy, electroencephalogram, non-linear analysis

## Abstract

Biomedical signals are measurable time series that describe a physiological state of a biological system. Entropy algorithms have been previously used to quantify the complexity of biomedical signals, but there is a need to understand the relationship of entropy to signal processing concepts. In this study, ten synthetic signals that represent widely encountered signal structures in the field of signal processing were created to interpret permutation, modified permutation, sample, quadratic sample and fuzzy entropies. Subsequently, the entropy algorithms were applied to two different databases containing electroencephalogram (EEG) signals from epilepsy studies. Transitions from randomness to periodicity were successfully detected in the synthetic signals, while significant differences in EEG signals were observed based on different regions and states of the brain. In addition, using results from one entropy algorithm as features and the k-nearest neighbours algorithm, maximum classification accuracies in the first EEG database ranged from 63% to 73.5%, while these values increased by approximately 20% when using two different entropies as features. For the second database, maximum classification accuracy reached 62.5% using one entropy algorithm, while using two algorithms as features further increased that by 10%. Embedding entropies (sample, quadratic sample and fuzzy entropies) are found to outperform the rest of the algorithms in terms of sensitivity and show greater potential by considering the fine-tuning possibilities they offer. On the other hand, permutation and modified permutation entropies are more consistent across different input parameter values and considerably faster to calculate.

## 1. Introduction

In a biomedical context, a signal describes a physiological state of a biological system that is part of a biological organism under investigation. The interpretation of those signals is not always trivial, due to either their nature, or the underlying physiological system that generates the signal. For this reason, feature extracting methods have been developed in order to uniquely identify different signal characteristics [1]. 

Traditional feature extracting methods can be mainly divided into two large categories; one originating from time-domain analysis and one from frequency-domain analysis [2]. Examples in the first category include autoregressive modelling (AR), cepstrum analysis and linear predictive modelling (LPC) while the second includes Fourier transform (FT) and Hilbert transform. A third category can be the combination of these two; in this way the advantages of both are combined or limitations are cancelled out. An example in this category is the wavelet transform (WT).

The study of non-linear dynamical analysis falls under the category of time-domain analysis and is well-suited to the analysis of biomedical signals as a result of the complex dynamics contained therein. Among the different non-linear analysis methods, entropy algorithms have found many applications in the context of biomedical signal analysis [3]. Nevertheless, in many cases these entropy algorithms are applied to the analysis of signals without an a priori, thorough understanding of what they measure.

Entropy methods have showed great potential in the analysis of electroencephalogram (EEG) signals—recordings of the electrical activity of the human brain. This is mostly due to the high complexity of the human brain and the non-linear interactions between the neurons, which translates into a signal whose dynamics might be characterised in more detail using entropy algorithms. Research in this area could help characterise in more detail the EEG signals in, for example, epilepsy, Alzheimer’s disease (AD), other forms of dementia, Parkinson’s disease (PD), and different kinds of sleep disorders, which that could help us obtain a greater understanding of these pathological conditions and, eventually, be use them for early diagnosis. 

Over the years many different entropy algorithms have been introduced in the literature, all of them relying on the idea of detecting chaotic or regular behaviour in the biomedical signals. Two large families of those, which are covered in our study, are the algorithms based on Shannon’s entropy and those based on embedding. Permutation entropy (PEn) and modified permutation entropy (MPEn) belong to the former group, while sample entropy (SEn), quadratic sample entropy (QSEn) and fuzzy entropy (FEn) belong to the latter family. 

PEn was first introduced by Bandt and Pompe [4]. It is based on calculating Shannon’s entropy of fixed length partitions of the signal, which are grouped in bins based on the frequency of appearance of their permutation pattern. Several studies using PEn have focused on classifying epileptic and healthy EEG data. Cao et al. [5] studied three patients with epileptic events by recording intracranial EEGs and by using PEn to detect any dynamic change. They found that PEn drops suddenly when a seizure occurs and then immediately starts to increase gradually until it reaches the normal values again. Nicolaou and Georgiou [6] trained a support vector machine (SVM) algorithm with PEn features extracted from healthy and epileptic EEG data, and succeeded in predicting between the two with a classification accuracy of over 90%. Staniek and Lehnertz [7] studied 15 subjects diagnosed with epilepsy by using EEG data and combining PEn and Transfer Entropy. Their study proved the potential of identifying the epileptogenic area without even recording an actual epileptic event.

More recently, modified permutation entropy (MPEn) has been introduced as a modified version of PEn in which the presence of equal signal values is handled differently [8]. It has been used to distinguish between young and elderly heart rate variability (HRV) signals, and between signals of subjects with severe congestive heart failure. Compared to PEn, it has been found to improve greatly, the ability to distinguish between signals in cases when the occurrence of equal values is an intrinsic property of the signal (i.e., the HRV signal) or when the digitisation resolution is low. In addition, MPEn has been used to distinguish between simulated EEG signals of normal and epileptiform types created using a single neural mass model [9]. Comparative statistical results suggested the better performance of MPEn compared to the conventional PEn algorithm.

A third approach for quantifying the complexity of a time series is SEn, which is a modified improvement of approximate entropy (ApEn) and was introduced by Richman and Moorman [10]. The authors, in this seminal study, performed tests on cardiovascular data, including heart rate and chest volume, and pointed to the increased statistical consistency of SEn on such data. Continuing on cardiovascular physiology applications, Alcaraz and Rieta [11] performed a wide study with SEn for the non-invasive analysis of atrial fibrillation (AF), in which they used typical surface electrocardiography data and presented robustness in detecting spontaneous AF termination, and variations of onset and rhythm maintenance, suggesting a useful tool for the non-linear quantification of cardiac dynamics in general. Moreover, Zhang and Zhou [12] proposed a novel technique for discriminating the onset of surface electromyography (EMG) activity from random background spikes or noise using an implementation of SEn and proved an effective reduction of latency time compared to other methods, with promising applications to any application involving EMG and muscle activity.

Despite its wide use, SEn is now often combined or replaced with QSEn. This method was introduced by Lake [13] as an improvement that is less dependent on its input parameters, as it includes a normalisation with respect to them. QSEn was applied to normal and AF cardiac rhythm signals to demonstrate the algorithm’s improvements over SEn [13]. After its introduction, it has also been applied to short biomedical signals, such as the blood pressure signal recorded using ambulatory blood pressure monitoring. Statistical results in the study revealed the ability of QSEn to distinguish between control and pathological groups while SEn and ApEn could not [14]. Relatively short signals have also been successfully characterised using QSEn for the classification of AD based on EEG recordings [15]. QSEn has shown a statistically significant increase in regularity of AD patients’ EEG signals in complete concordance with SEn and ApEn but for a greater range of input parameters, while the optimal parameter values have been found to be outside of the other algorithms’ ranges. Finally, QSEn has been recently used to distinguish between sleep stages (quiet and active sleep) based on the HRV of newborn infants [16], and also to classify between calm and negative stress based on EEG recordings with results showing great potential [17].

The modification of the original SEn algorithm has also led to the introduction of another entropy measure, which is now widely known as fuzzy entropy (FEn). This recent embedding type of entropy differs in the way it calculates the similarity degree of signals and could be considered as a more analytical, accurate, and consistent (i.e., under different settings) version of SEn [18]. FEn has been applied in EEG studies of AD, in which it has shown greater robustness to noise, better distinguishing ability between AD and control signals, and better classification accuracy compared to SEn and ApEn [3,19]. In addition, its suitability even for short biomedical signals and outperformance of SEn and ApEn has been demonstrated by successfully tracking qualitative changes in surface EMG signals during different motion tasks, during muscle fatigue, and during a 45-day head-down bed-rest experiment in simulated microgravity [20,21,22]. Finally, FEn has also recently shown its potential in biometrics applications according to a study on EEG-based person authentication. The authors have shown that using FEn as a feature results in classification accuracy greater than 87.3%, while only two frontal electrodes are required. The study was heavily based on the algorithm’s robustness to noise and sensitivity to different levels of signal randomness which eventually aid in the reduction of electrodes and the improvement of classification rates simultaneously [23].

In spite of all the previous work done with these entropy metrics, it is necessary to understand them in greater detail, in particular the relationship of entropy to relevant signal processing concepts. To that end, we created different synthetic signals with different signal characteristics that are widely encountered in signal analysis, particularly in the biomedical context, and analysed them with the five entropy algorithms aforementioned. This was done to reveal the relationship of the different entropy algorithms with those signal characteristics and the ability of each of the algorithm to detect changes in the properties of the signals. Subsequently, and to test the possible usefulness of these entropy algorithms in the classification of biomedical signals in a clinical context, we applied the algorithms to the analysis of two different epileptic EEG data sets. The first EEG data set contained two groups of data: One with surface EEG segments recorded from healthy control subjects with eyes open or closed, and one with intracranial epileptic EEG segments recorded during ictal or interictal periods and at different brain locations. The second EEG data set contained interictal segments corresponding to focal or non-focal channels. We hypothesised that it would be possible to observe statistically significant differences in the means of populations belonging to different classes of signals by means of the entropy algorithms. Finally, we also attempted to predict the class to which the EEG signals belonged to using their entropy measures as features. In this way, a thorough theoretical and practical comparison of the five entropy algorithms is provided.

The structure of the paper is as follows. Section 2 contains a detailed description of the entropy algorithms to be tested, the synthetic signals created to that aim, and the EEG data used to evaluate the possible clinical usefulness of the different entropy metrics. Exhaustive results are presented in Section 3, which is followed by the discussion and, finally, the conclusions extracted from our study.

## 2. Materials and Methods 

### 2.1. Entropy Algorithms

#### 2.1.1. Permutation Entropy

Shannon’s entropy describes the average rate of information associated with a stochastic source of data and, therefore, could be a very good measure of the complexity of a time series. However, it does not account for the temporal order of successive signal samples. PEn partitions the signal in sequences of length *n* and then encodes these based on their ranks. In that way, a limited number of possible permutations are identified and it becomes possible to observe that certain patterns appear more often than others. To calculate PEn, the classic Shannon’s entropy formula is applied, based on the frequency of appearance of these permutation patterns. In this study, we implemented the original algorithm from Bandt and Pompe [4] and tested it for *n* = [3, 4, 5, 6]. Those values follow the recommendations regarding *n* needing to be greater than 3, and *n* < *N*!, where *N* is the number of samples of the time series being analysed [4].

#### 2.1.2. Modified Permutation Entropy

The only intrinsic limitation of the PEn algorithm relies on the occurrence of equal values in the *n* length partitions. Usually, in PEn, those are assigned with different ranks based on their order of appearance in the signal. Other times a small white noise is added to the signal so that equalities are resolved in a random way, or the signal is down-sampled in different ways [24]. However, when the occurrence of equal values is an intrinsic feature of the biomedical signal itself, MPEn is a better approach to PEn. In MPEn, equalities are also included in the possible permutation patterns, resulting in more possible patterns (approximately *n* − 1 times more). In our study, we used the original MPEn introduction study from Bian et al. [8] to develop the algorithm and tested it for *n* = [3, 4, 5]. The choice of these values for *n* was a compromise between using similar ones than with PEn and not having a number of permutation patterns that would exceed the total number of samples in the time series being analysed (for *n* = 6, the upper bound of permutation patterns would be 4051 [8]). 

#### 2.1.3. Sample Entropy

SEn is a widely used entropy algorithm that falls under the category of embedding entropies, which attempt to quantify the complexity of a time series by comparing the series with a delayed version of itself [25]. SEn is an improvement over ApEn in which strong dependencies on signal length and algorithm input parameters are reduced [10]. As a result, SEn is independent of the series length, is more consistent, and requires half the time to be calculated [26]. However, choosing the right input parameter values is not trivial, since there is a trade-off between different options [27]. We used [26] to implement the algorithm and tested 8 different input parameter combinations (*m* = [1, 2], *r* = [0.1, 0.15, 0.2, 0.25]), as suggested by Richman and Moorman in their seminal paper introducing this entropy algorithm [10].

#### 2.1.4. Quadratic Sample Entropy

To overcome the previously mentioned limitations associated with SEn, QSEn has been introduced [13]. QSEn attempts to convert the measured conditional probability to a probability density by normalising the number of matches to the volume of the matching region ${\left(2r\right)}^{m}$. This leads to the direct calculation of QSEn based on the original SEn algorithm as follows:

(1) QSEn(m, r, N)=SEn(m, r, N)+log(2r) ,

Advantages over SEn are related to the input parameter *r*. In QSEn, there is no constraint on values it can get, while also, due to the normalisation, it offers the ability to directly compare entropies of different types of signals, even when different *r* values have been used [13,15]. In our study, we used the original algorithm implementation and tested 8 different input parameter combinations (*m* = [1, 2], *r* = [0.2, 0.4, 0.6, 0.8]). The values of *m* used in the computation of QSEn are similar to those used with SEn to make comparisons straightforward. The range of values for *r* was chosen to test one of the advantages of QSEn; namely, it being capable of producing a reliable measure of entropy for a wider range of values of *r* than SEn [13].

#### 2.1.5. Fuzzy Entropy

FEn is based on SEn but further extends its definition. This is done by generalising the way the similarity is calculated between vectors. In SEn and QSEn, the similarity is calculated on a binary basis, using a Heaviside function as below:

(2) $$D\left(r,\;d_{ij,m}\right)=\theta \left(r-d_{ij,m}\right)=\{\begin{array}{c} 0,\;\;d_{ij,m}>r\\ 1,\;\;d_{ij,m}\leq r \end{array}\;,$$

In FEn, however, this is replaced with an exponential function:

(3) $$D\left(r,\;n,\;d_{ij,m}\right)=\exp\left(\frac{-{\left(d_{ij,m}\right)}^{n}}{r}\right)$$

This approach leads to an infinite number of possible similarity degree values instead of a two-state classification, while providing an extra input scaling parameter *n*. In our study, we implemented the algorithm from [20] and tested 24 input parameter combinations (*n* = [1, 2, 3], *m* = [1, 2], *r* = [0.1, 0.15, 0.2, 0.25]). The choices of values for *m* and *r* are based on FEn being a modification of SEn, while the different values of *n* allow testing similarity using different exponential shapes as in [3].

### 2.2. Synthetic Signals

Ten different synthetic signals were generated to study and reveal the relationship between different signal characteristics and entropy algorithms. The characteristics considered are based on both general signal characteristics and those encountered particularly often in biomedical signals. All signals were created using a sampling frequency of 256 Hz and were 40 s long. The sampling frequency matches the standard one used in EEG recording devices. The duration of 40 s was chosen to include 4 different windows of 10 s in which the characteristics of the signal either changed or became gradually more dominating. More particularly, the signals were:

#### 2.2.1. Constant Amplitude Chirp Signal

**Figure 1:**
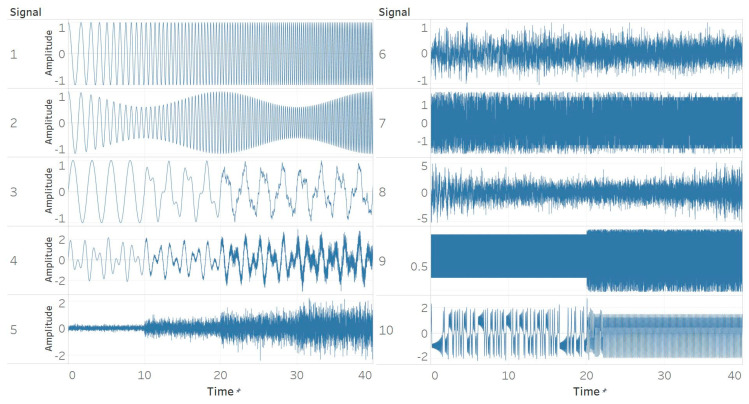
Synthetic signals. (1) Constant amplitude chirp signal; (2) modulated amplitude chirp signal; (3) signal with increasing number of harmonics; (4) quasi-periodic signal with increasing noise power; (5) white Gaussian noise (WGN) of increasing power; (6) WGN of increasing bandwidth; (7) MIX process; (8) autoregressive (AR) process; (9) logistic map; (10) Lorenz system.

The first signal is created with a pure sinusoid with constant amplitude and a linear sweep of frequency from 0.5 to 5 Hz from *t* = 0 s to *t* = 40 s (Figure 1). This is to test how changes in the frequency of a pure sinusoid affect the entropy algorithms while keeping the amplitude constant.

#### 2.2.2. Modulated Amplitude Chirp Signal

The second signal is obtained by applying an amplitude modulation to the first signal. Specifically, a cosine of 0.05 Hz frequency, amplitude of 0.25 and offset of 0.75 was applied (Figure 1). This test extends the first one and includes amplitude changes in a sinusoidal chirp structure.

#### 2.2.3. Signal with Increasing Number of Harmonics

The third signal consists of four different parts with a different number of sinusoidal harmonics. We used 1, 2, 5 and finally 7 harmonics respectively. The harmonics’ frequencies were spaced logarithmically between 0.4 Hz (base frequency) and 100 Hz (which is the upper boundary of the gamma EEG band) while their amplitudes ranged between 0.5 and 0.005 respectively (Figure 1). This test is to examine the effect of the increasing frequency content of a signal.

#### 2.2.4. Quasi-Periodic Signal with Increasing Noise Power

For this test, we wanted to examine the effect of increasing levels of noise power which is present in quasi-periodic signals. For this purpose, we added two sinusoids of 0.61 and 1 Hz and modulated them with a low-frequency modulator (0.05 Hz), low amplitude (0.1), and offset of 1. Then, we segmented the signal by adding white Gaussian noise (WGN) of magnitudes 0, 0.1, 0.3, and 0.5 to the four time windows (Figure 1).

#### 2.2.5. White Gaussian Noise (WGN) of Increasing Power

The fifth signal is made of four pure WGN realisations of powers 0.1, 0.3, 0.5 and 0.7 with respect to the time windows (Figure 1). This tests the effect of increasing noise power given the same underlying signal structure (WGN).

#### 2.2.6. White Gaussian Noise (WGN) of Increasing Bandwidth

To extend the previous test and examine the effect of noise bandwidth, we generated a single realisation of WGN and then three low-pass filters with cut-off frequencies of 30, 60 and 100 Hz for the three first parts of the signal, while the final part was unfiltered (maximum bandwidth). We used finite impulse response (FIR) filters designed using a Hamming window and order of 425. The upper boundary of 100 Hz again, represents here, the upper limit of the EEG gamma band (Figure 1).

#### 2.2.7. MIX process

For this test, we aimed at analysing differences between stochastic and periodic deterministic signals, and thus generated a synthetic signal using the definition of a MIX process and the relevant procedure followed by previous studies [28]. First, we define a periodic sequence *x* as:

(4) $$x_{k}=\;\sqrt{2}\;\sin\left(\frac{2\pi k}{12}\right)\;,$$

with *k* representing the number of samples. Then, we define a uniformly distributed sequence from $-\sqrt{3}$ to $+\sqrt{3}$ using a uniform random generator. Then, we also define a Bernoulli random variable *z*, which is equal to 1, with probability *p,* and equal to 0, with probability 1 − *p*. The signal is then defined as:

(5) MIX=(1−z)x+z ,

The *z* parameter randomly either favours *x* or *y* signal (periodic or random) and its value is controlled by probability *p*. To switch from a stochastic to periodic deterministic sequence, we swept *p* linearly from 0.9 to 0.1 with time (Figure 1).

#### 2.2.8. Autoregressive (AR) process

The eighth signal was generated using an AR process of order 1 (AR(1)). The coefficient of the first (and only in our case) past value *p* was swept linearly from +0.9 to −0.9 in order to model the transfer of coloured noise energy from low to high frequencies, and therefore study the effect of the spectral content of coloured noise (Figure 1). *P* value of 0 corresponds to WGN. To counter the initialisation issue of the algorithm, we discarded the first 100 samples [28].

#### 2.2.9. Logistic map

We extended the approach used in the MIX process and included a logistic map as well, which uses a similar approach to that of an AR process. The equation used to create the signal is [28]:

(6) $$x_{k}=\mu x_{k-1}\;\left(1-x_{k-1}\right)\;,$$

Specifically, we used two segments of a logistic map with model parameter *μ* of 3.55 and 3.8 respectively. Using *μ* = 3.55 produces a signal that oscillates between 8 values, while using *μ* = 3.8 incorporates a chaotic behaviour (Figure 1). We used 0.3 as a starting point and discarded the first 100 samples. The ability of entropy algorithms to detect the change in *μ* parameter, which models the transformation from a periodic to a chaotic behaviour, is examined here.

#### 2.2.10. Lorenz system

Finally, we modelled a non-linear system called Lorenz attractor, which is described by the following differential equations [28]:

(7) $$\dot{x}=\sigma \;\left(y-x\right)\;\dot{y}=x\;\left(\rho -z\right)-\;y\;\dot{z}=xy-\beta z\;,$$

where *σ*, *β*, and *ρ* are system parameters. We segmented the signal in half using two different sets of parameter values. The first set was with *σ* = 10, *β* = 8/3, and *ρ* = 28, and is characterised by chaotic behaviour. The second set was with *σ* = 10, *β* = 8/3, and *ρ* = 99.96, and produced a torus knot. The coordinate *x* was used as our synthetic time series. Both parts were normalised so that their standard deviation equals 1 (Figure 1).

### 2.3. Electroencephalogram and Epilepsy Studies (I)

The first EEG data set in this study was acquired by Andrzejak et al. [29] at the University of Bonn. It consists of five different data sets of 100 single-channel EEG segments of 23.6 s duration each. The segments are parts of multichannel EEG recordings and were selected to fulfil two different criteria. Firstly, they had to be free of any unwanted eye or muscle movement artefacts, and for that purpose, they were visually inspected. Secondly, a weak stationarity criterion was applied to ensure fluctuations of amplitude deviations, and frequency remained inside a well-defined distribution. This process is explained in more detail in the original study. As part of the original study and in terms of instrumentation characteristics, a 128-channel amplifier system was used for all recordings, along with a 12-bit analogue-to-digital converter (ADC) with a sampling frequency of 173.61 Hz. In our study, we further applied a digital FIR bandpass filter of order 425, designed using a Hamming window. The passband chosen was between 0.5 Hz and 40 Hz to avoid DC components and 50 Hz mains noise.

The first subset of signals (group A) contains EEG data from five healthy volunteers recorded with surface electrodes placed according to the standard 10–20 international system and during a relaxed, awake state. The first data set was recorded with eyes open (data set 1) while the second one was with eyes closed (data set 2). The second subset of EEG signals (group B) contains three data sets (denoted as data set 3, data set 4, and data set 5) with intracranial EEG signals from five epilepsy patients who have had surgical resection of one of the hippocampal formations that have been identified as epileptogenic zones. After this surgery, they had achieved complete seizure control. However, the data were recorded before the surgery, at the diagnostic stage. More specifically, data sets 3 and 4 contain data recorded at seizure-free intervals, with data set 3 recorded at the hippocampal formation of the opposite hemisphere and data set 4 at the epileptogenic zone. In contrast, data set 5 was chosen to contain only seizure activity from all sites exhibiting ictal activity. For all our analysis purposes, we considered data sets 1 and 2 in one group (group A), and data sets 3, 4, and 5 in another (group B).

### 2.4. Electroencephalogram and Epilepsy Studies (II)

The second EEG data set used in this study was published by Andrzejak, Schindler, and Rummel [30] and contains recordings that were made on five epilepsy patients for diagnostic purposes. An epilepsy surgery was suggested for them, as they all had longstanding pharmacoresistant temporal lobe epilepsy. To localise the seizure onset zones, intracranial EEG signals were recorded with a sampling frequency of 512 Hz. The original data set contains 7500 signal pairs, out of which 3750 pairs originate from focal channels and 3750 originate from non-focal channels. For each pair, we have one signal from the actual channel and one from a neighbouring channel. Each signal is 20 s long, resulting in 10,240 samples. A pre-processing step through which a band-pass filter with cut-off frequencies Fc1 = 0.5 Hz and Fc2 = 150 Hz was applied to the data by the authors of the original study. As part of this study, though, we only used 500 signal pairs out of 7500. This smaller number was selected to match the number of EEG segments analysed in the first data set used in study but also to reduce computational load. 

The data set is divided into two classes. The first one, termed “focal EEG channels” contains EEG data from channels in which the first ictal EEG activity was detected [30]. The second one, termed “non-focal EEG channels” contains data from all other channels that did not experience the first ictal activity. In both cases, recordings were made during interictal periods and no seizure activity was included. For each of the 500 recordings, a signal pair was recorded. The first signal, termed as *x*, was the actual signal identified as focal or non-focal from the visual inspection, while the second signal, termed *y*, was chosen to be a signal from the neighbouring channels. In our study, we only used the *x* signals from both focal and non-focal channels.

### 2.5. Statistical Analysis and Classification

For statistical analysis purposes, we employed different hypothesis testing techniques. In the first epilepsy study and in group A (non-epileptic EEG with eyes open/closed), we used a Wilcoxon signed-rank test, since the two sets of data were dependent and the distributions, not normal. The dependency is attributed to the fact that the two data sets are essentially repeated measurements of the same subjects. In group B, we used a Kruskal–Wallis test, since the three sets of data were independent and distributions not normal. Accordingly, in the second epilepsy study, we used a Mann–Whitney U test, since the two sets of data were independent and distributions not normal. 

For classification purposes, we used a k-nearest neighbours (k-NN) algorithm with *k* = 3 and different combinations of entropy features. We chose the particular algorithm due to its combined simplicity, and good and indicative performance. To train the algorithm, we randomly used 90% of the data, while 10% was used for testing through a 10-fold cross-validation method. The size of the feature vector varied from 1 to 5. For sizes greater than 1, we excluded combinations that would contain the same algorithm with different input parameter settings. Therefore, a total of 5 features would correspond to one feature per entropy algorithm. Under these settings, we studied three different classification tasks; one in the surface EEG signals from [29], one in the intracranial signals from [29], and one in the second epilepsy study [30].

## 3. Results

### 3.1. Synthetic Signals

**Figure 2:**
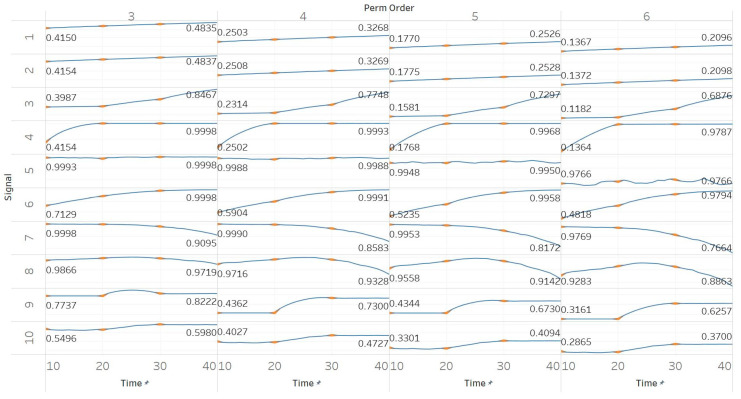
Synthetic signals entropy results using permutation entropy (Pen) with permutation order (Perm Order) *n* equal to 3, 4, 5, and 6. Numbers on the *y* axis correspond to the synthetic signals: (1) Constant amplitude chirp signal; (2) modulated amplitude chirp signal; (3) signal with increasing number of harmonics; (4) quasi-periodic signal with increasing noise power; (5) WGN of increasing power; (6) WGN of increasing bandwidth; (7) MIX process; (8) AR process; (9) logistic map; (10) Lorenz system. For each synthetic signal and figure, the numbers represent the first (at *t* = 10 s) and final (at *t* = 40 s) entropy values, while the orange dots correspond to the time stamps when the parameters from the synthetic signals change (*t* = 10/20/30 s).

PEn was calculated for all possible order values *n* = [3, 4, 5, 6] using 10 s windows with a 90% overlap between consecutive windows (results are shown in Figure 2). PEn is linearly affected by the change in the frequency of a pure sinusoid, yet this effect is unchanged if amplitude modulation is added to it. A similar but bigger (exponential) increase is observed in the event of adding harmonics to a signal. Next, increasing the noise power in a quasi-periodic signal quickly saturates the entropy to its maximum, while using a pure WGN signal already saturates to maximum entropy without significant changes over noise power changes. The increasing noise bandwidth, on the other hand, shows a logarithmic increase in entropy, again reaching the maximum possible value. A transition from randomness to deterministic periodicity, modelled through the MIX process, is associated with a decrease in entropy. In the AR(1) process, shifting from coloured noise to WGN and back, produced a concave function shape which proves the increased sensitivity of PEn to noise bandwidth. The MIX process shifts from quasi-periodicity to chaos in the middle of the signal and is successfully detected with an increase in PEn. The shift from chaotic behaviour to a form of periodic knot in the Lorenz system signal is again associated with an increase in PEn, a fact that suggests the thin line between entropies of periodic elements and regular chaotic elements in a signal. It is worth noting, though, that our implementations of both the MIX process and the Lorenz system appear (based on a visual inspection) more regular in their first half; therefore, explaining those results.

**Figure 3:**
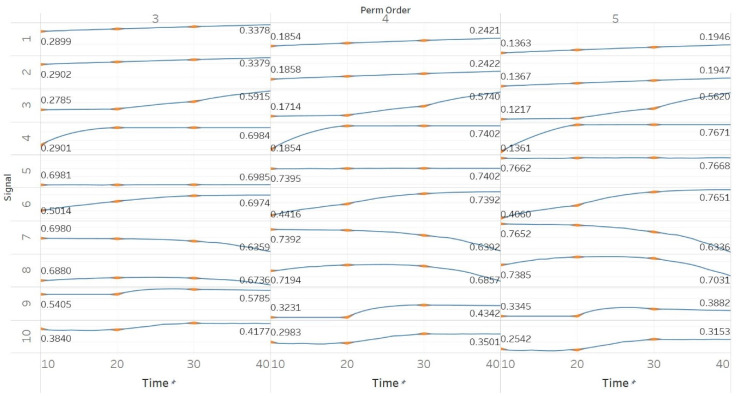
Synthetic signals entropy results using modified permutation entropy (MPEn) with permutation order (Perm Order) *n* equal to 3, 4, and 5. Numbers on the *y* axis correspond to the synthetic signals: (1) Constant amplitude chirp signal; (2) modulated amplitude chirp signal; (3) signal with increasing number of harmonics; (4) quasi-periodic signal with increasing noise power; (5) WGN of increasing power; (6) WGN of increasing bandwidth; (7) MIX process; (8) AR process; (9) logistic map; (10) Lorenz system. For each synthetic signal and figure, the numbers represent the first (at *t* = 10 s) and final (at *t* = 40 s) entropy values, while the orange dots correspond to the time stamps when the parameters from the synthetic signals change (*t* = 10/20/30 s).

MPEn was tested for *n* = [3, 4, 5] using 10 s windows with a 90% overlap (results are shown in Figure 3). Results in MPEn are in complete accordance with those of PEn. Nevertheless, it is worth noting the fact that the relevant saturation to maximum entropy now only happens approximately at an absolute value of 0.7 rather than one. This is explained by the definition of MPEn and Shannon entropy, where an almost equal number of appearances of all permutation patterns is impossible, as patterns that include equalities are less likely to happen in a noisy signal.

**Figure 4:**
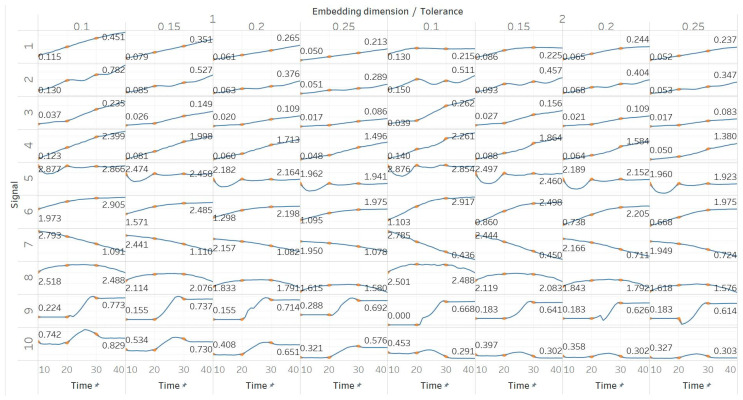
Synthetic signals entropy results using sample entropy (Sen) with embedding dimension *m* equal to 1 and 2 and tolerance *r* equal to 0.1, 0.15, 0.2, and 0.25. Numbers on the *y* axis correspond to the synthetic signals: (1) Constant amplitude chirp signal; (2) modulated amplitude chirp signal; (3) signal with increasing number of harmonics; (4) quasi-periodic signal with increasing noise power; (5) WGN of increasing power; (6) WGN of increasing bandwidth; (7) MIX process; (8) AR process; (9) logistic map; (10) Lorenz system. For each synthetic signal and figure, the numbers represent the first (at *t* = 10 s) and final (at *t* = 40 s) entropy values, while the orange dots correspond to the time stamps when the parameters from the synthetic signals change (*t* = 10/20/30 s).

SEn was calculated using 10 s windows with a 90% overlap for values of embedding dimension *m* = [1, 2] and tolerances *r* = [0.1, 0.15, 0.2, 0.25] times the standard deviation (results are shown in Figure 4). Results from most signals are almost identical to those obtained from PEn and MPEn, although some differences can be observed. SEn values increase with noise power of a quasi-periodic signal with a linear response without early saturation. Saturation is also delayed in the increasing noise bandwidth signal. The gradient shift in the AR(1) process is also better reflected in the shape of the resulting entropy curve. SEn also detects the step change from quasiperiodicity to chaos in the logistic map signal as well, but with significantly increased step increase compared to previous algorithms. Finally, SEn is also detecting the Lorenz system transition at *t* = 20 s in a similar way but with a smaller step increase for *m* = 1. For *m* = 2, that increase only happens between *t* = 20 s and *t* = 30 s, where the moving window contains both parts of the signals’ realisations. This result is in line with relevant results from the literature [28]. It is also worth noting, additionally, that the entropy values return to a lower level after *t* = 30 s, suggesting an increased entropy for the chaotic part for *m* = 2 only. 

**Figure 5:**
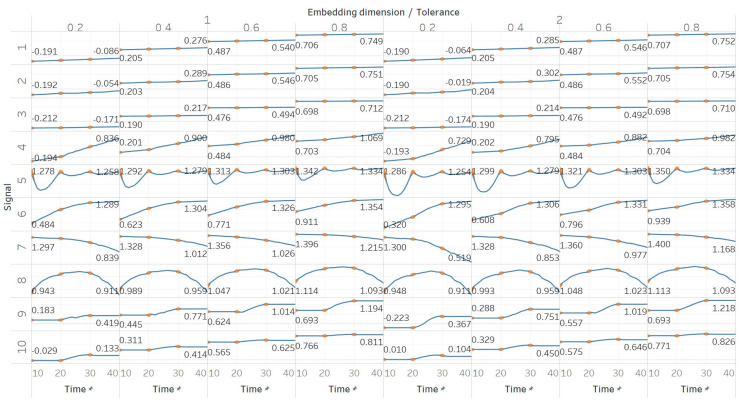
Synthetic signals entropy results using quadratic sample entropy (QSEn) with embedding dimension *m* equal to 1 and 2 and tolerance *r* equal to 0.2, 0.4, 0.6, and 0.8. Numbers on the *y* axis correspond to the synthetic signals: (1) Constant amplitude chirp signal; (2) modulated amplitude chirp signal; (3) signal with increasing number of harmonics; (4) quasi-periodic signal with increasing noise power; (5) WGN of increasing power; (6) WGN of increasing bandwidth; (7) MIX process; (8) AR process; (9) logistic map; (10) Lorenz system. For each synthetic signal and figure, the numbers represent the first (at *t* = 10 s) and final (at *t* = 40 s) entropy values, while the orange dots correspond to the time stamps when the parameters from the synthetic signals change (*t* = 10/20/30 s).

QSEn was tested using the same embedding dimensions *m* = [1, 2], window size and overlap as SEn, but tolerances of *r* = [0.4, 0.6, 0.8, 1] (results are shown in Figure 5). In general, we observe a complete match of results with those of SEn, apart from the fact that QSEn maintains consistency between *m* = 1 and *m* = 2 in the Lorenz system results. There is a better consistency of results in terms of absolute entropy values between different parameter combinations. This proves the relevant advantages of QSEn described earlier.

**Figure 6:**
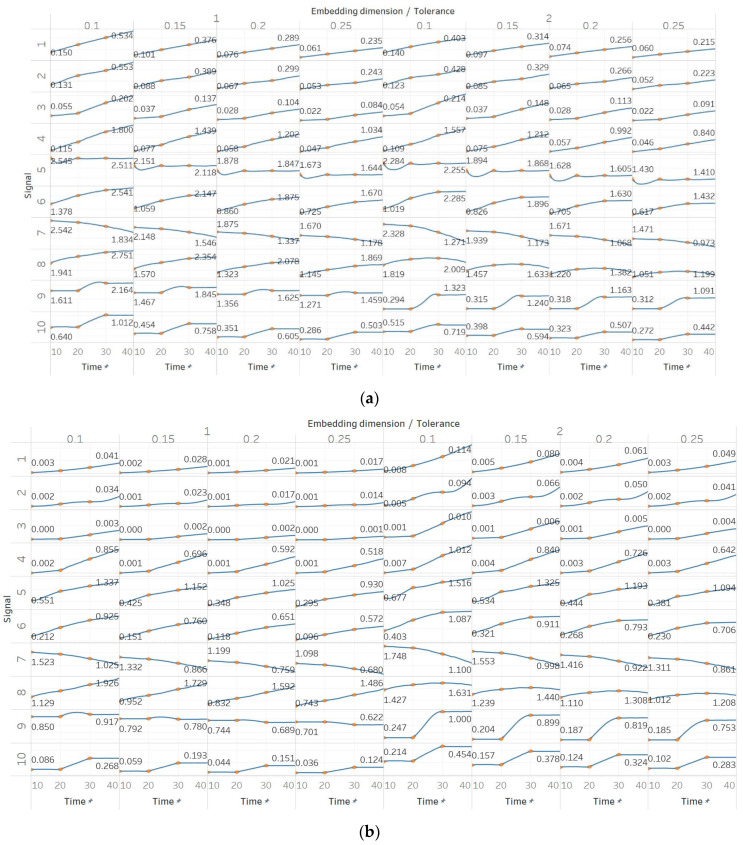

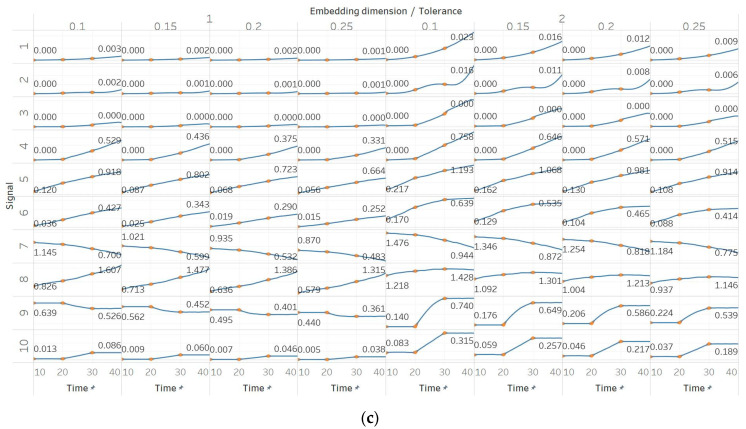
Synthetic signals entropy results for fuzzy entropy (Fen) with embedding dimension *m* equal to 1 and 2, and tolerance *r* equal to 0.2, 0.4, 0.6, and 0.8, and: (**a**) *n* = 1; (**b**) *n* = 2; (**c**) *n* = 3. Numbers on the *y* axis correspond to the synthetic signals: (1) Constant amplitude chirp signal; (2) modulated amplitude chirp signal; (3) signal with increasing number of harmonics; (4) quasi-periodic signal with increasing noise power; (5) WGN of increasing power; (6) WGN of increasing bandwidth; (7) MIX process; (8) AR process; (9) logistic map; (10) Lorenz system. For each synthetic signal and figure, the numbers represent the first (at *t* = 10 s) and final (at *t* = 40 s) entropy values, while the orange dots correspond to the time stamps when the parameters from the synthetic signals change (*t*=10/20/30 s).

In FEn, we used the same window size (10 s) and overlap (90%) than for the other entropy algorithms, and input parameters of *n* = [1, 2, 3], *m* = [1, 2] and *r* = [0.1, 0.15, 0.2, 0.25], resulting in 24 different combinations (results are shown in Figure 6). In general, we observed similar results to those of SEn and QSEn, with main differences happening for *n* = 2 or *n* = 3. In those cases, FEn produces very small values, while at the same time appears to be more sensitive to signal changes. For example, values for a constant amplitude chirp signal increase exponentially, rather than linearly, while, in the modulated version of it, the modulation is also reflected in the entropy as a small oscillation superimposed on the expected linearly increasing curve. Impressively, using *n* = 2 or *n* = 3 also resulted in a linear increase in FEn values for the increasing noise power signal. Finally, it is worth noting that FEn performed worse on the AR(1) process for most combinations, since a concave function shape was not observed in this case. In the logistic map signal, FEn provided varying results based on the parameter combination, while in the Lorenz system showed a consistent step increase across all possible parameter combinations.

### 3.2. Electroencephalogram and Epilepsy Studies (I)

**Figure 7:**
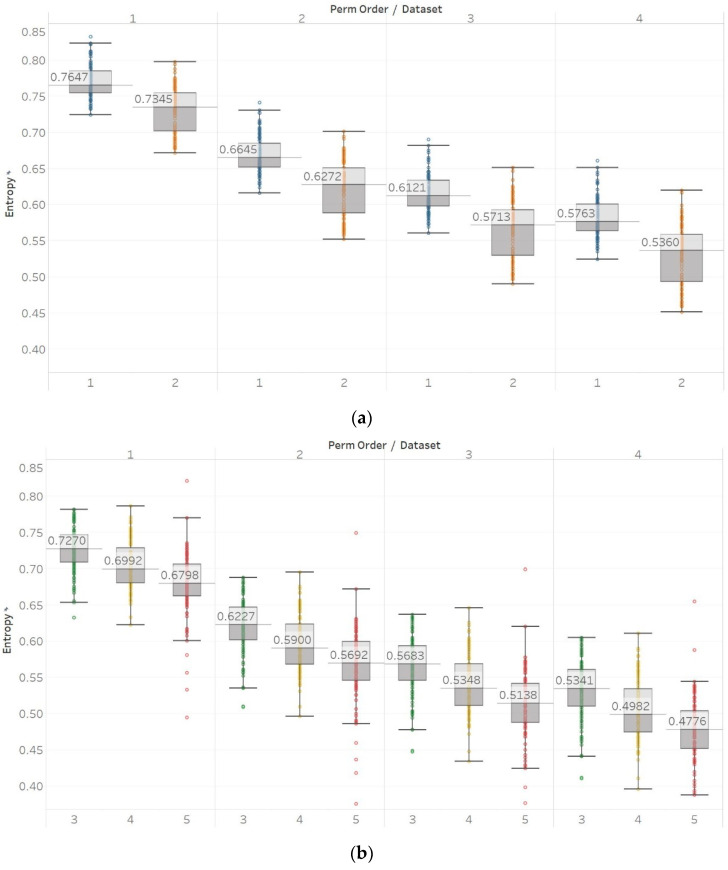
PEn results of electroencephalogram and epilepsy study (*I*): (a) group A; (b) group B. Data sets: (1) Eyes open healthy EEG; (2) Eyes closed healthy EEG; (3) Opposite hemisphere epileptic EEG; (4) Epileptogenic zone epileptic EEG; (5) Seizure EEG.

For PEn, a difference between the means of data set 1 and data set 2 (group A) could be observed (Figure 7). This difference is consistent among all values of permutation order *n*, with data set A resulting in higher entropy values than dataset B. This observation is explained by the *alpha* rhythm appearance in the event of closing the eyes (data set 2). *Alpha* rhythms are generally more regular and slower (10 Hz) and, therefore, could be associated with lower values of PEn [6]. Accordingly, for the intracranial EEG recordings, higher entropy values were obtained. More specifically, in data set C, we observe the highest values, as it is made of data recorded from the opposite hemisphere of the epileptogenic zone; therefore, maintaining the irregular behaviour of a normal EEG. In contrast, data set D produces slightly lower values, as the epileptogenic zone tends to produce more defined signal patterns, while, data set E produces even lower entropy values, as it includes epileptic seizures in the EEG segments, which are generally more regular and repeating patterns in terms of their rank sequence [6]. Finally, all results significantly reject the hypothesis (p≪0.01) for both groups and all values of *n*. To further examine for pairs of distributions in group B, Mann–Whitney U tests were also performed for each possible pair of data sets. Results reject the hypothesis of equal medians for all values of *n* and all three pairs of data sets (Supplementary Material, Tables S1–S3).

**Figure 8:**
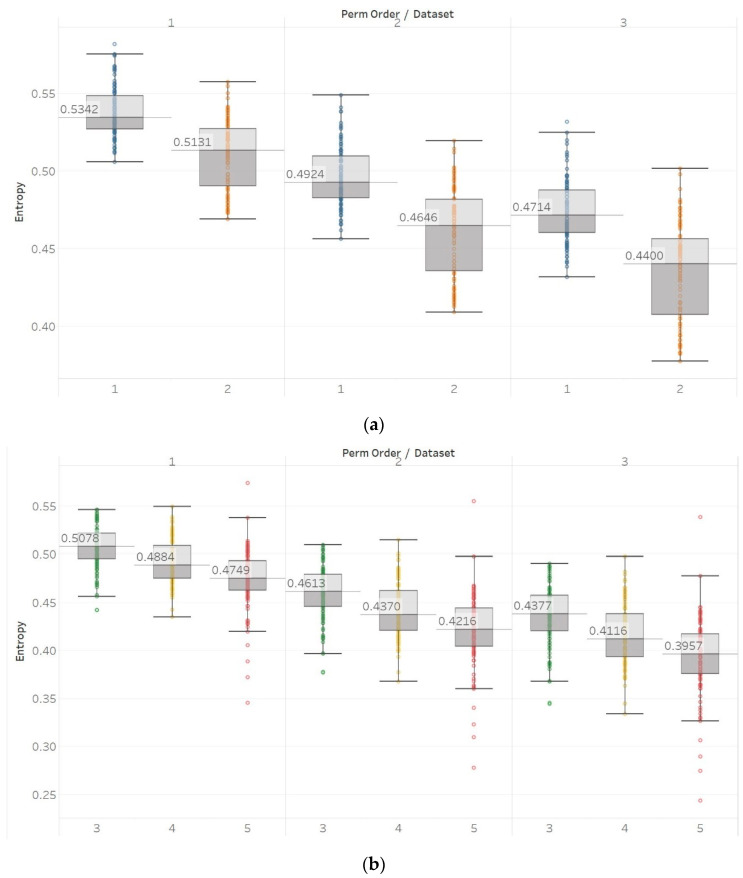
MPEn results of electroencephalogram and epilepsy study (*I*): (**a**) Group A; (**b**) group B. Data sets: (1) Eyes open healthy EEG; (2) wyes closed healthy EEG; (3) opposite hemisphere epileptic EEG; (4) epileptogenic zone epileptic EEG; (5) seizure EEG.

Similar results were obtained for MPEn, with the only relevant difference being the increased robustness to changes in the algorithm’s input parameter *n* (Figure 8). This robustness is accounted by the extension of the algorithm that takes into account equalities in a more generalised way [8]. Statistical tests results are identical to results from PEn tests (Supplementary Material, Tables S1–S3).

**Figure 9:**
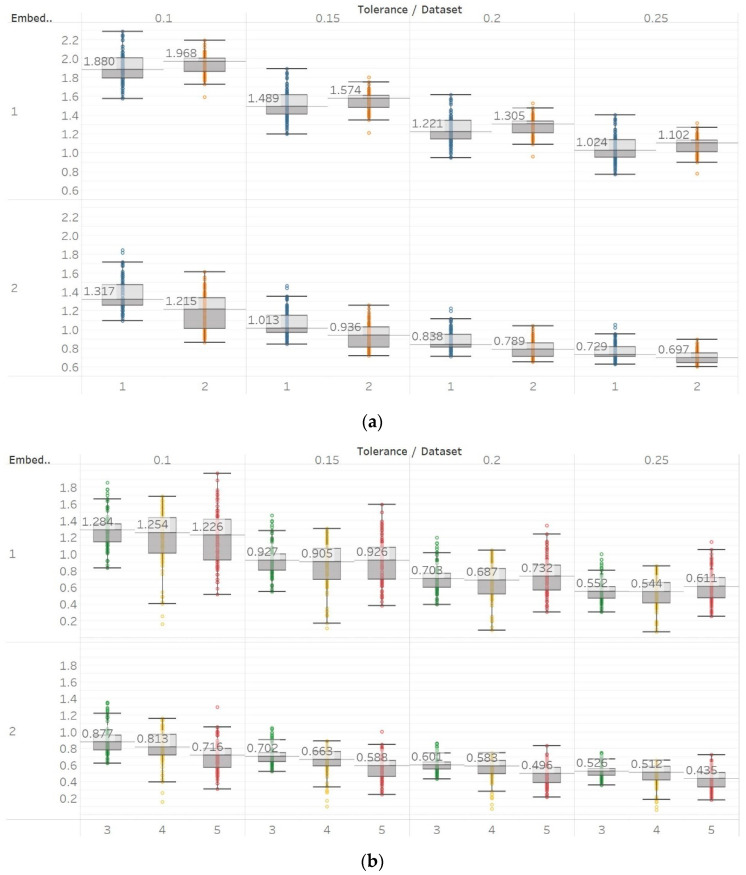
SEn results of electroencephalogram and epilepsy study (*I*): (**a**) Group A; (**b**) group B. Data sets: (1) Eyes open healthy EEG; (2) eyes closed healthy EEG; (3) opposite hemisphere epileptic EEG; (4) epileptogenic zone epileptic EEG; (5) seizure EEG.

On the other hand, in the case of Sen, results were similar to the previous ones but only using embedding dimension *m* = 2 (Figure 9). Using *m* = 1 results in Sen being higher in eyes closed compared to eyes open healthy EEG. It is worth noting, though, that the distribution of data set 1 contains both the highest and lowest values compared to data set B for all values of *r.* Additionally in group B, although the epileptogenic zone produces slightly less entropy than the opposite hemisphere, seizure EEG segments produce higher entropy values than the rest. Therefore, results suggest that *m* = 2 is a more suitable choice to characterise EEG signals than *m* = 1. In group A, all differences observed were statistically significant (*p* ≪ 0.01) for *m* = 2 and not significant for *m* = 1. In group B, using *m* = 1 did not provide statistically significant results between any of the possible pairs of data sets examined under the Mann–Whitney U tests. On the other hand, using *m* = 2 resulted in statistically significant differences between seizure and non-seizure data sets for all values of *r*, but not between data sets in the non-seizure group (Supplementary Material, Tables S1–S3).

**Figure 10:**
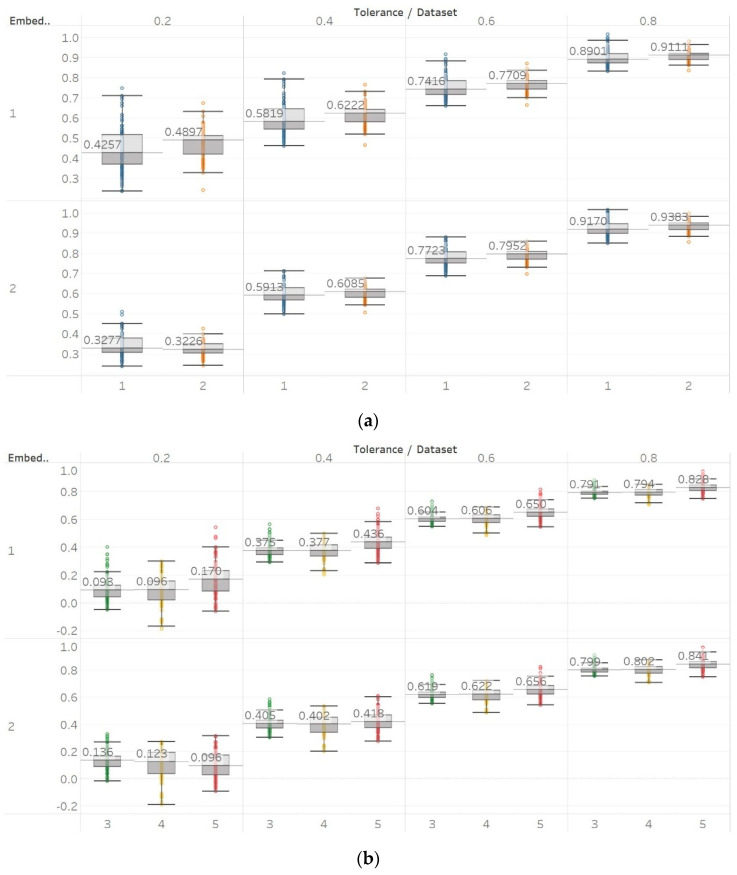
QSEn results of electroencephalogram and epilepsy study (*I*): (**a**) Group A; (**b**) group B. Data sets: (1) Eyes open healthy EEG; (2) eyes closed healthy EEG; (3) opposite hemisphere epileptic EEG; (4) epileptogenic zone epileptic EEG; (5) seizure EEG.

With QSEn, we aimed at examining larger tolerance values, and therefore, tested the algorithm for *m* = [1, 2] and *r* ranging from *r* = 0.4 to *r* = 1. Results for *m* = 1 are completely consistent with results for SEn with *m* = 1 (Figure 10). However, using *m* = 2 still produces results in accordance with *m* = 1, except for a single parameter combination of *m* = 2 and *r* = 0.2. On the other hand, as expected, increasing the algorithm tolerance value *r* also increases the entropy values (in this entropy algorithm only) as it includes an additive term which is proportional to *r* (log2r) [13,15]. Nevertheless, an interesting observation of QSEn results is that all standard deviations of populations are less affected by differences between EEG segments and input parameter values, while also staying at a significantly low level. In group A, observed differences are only statistically significant (p≪0.01) for *m* = 1, or *m* = 2, and *r* = 0.8 while in group B, differences are only significant between seizure and non-seizure data sets for all combinations except for *m* = 2 and *r* = [0.2, 0.4] (Supplementary Material, Tables S1–S3).

**Figure 11:**
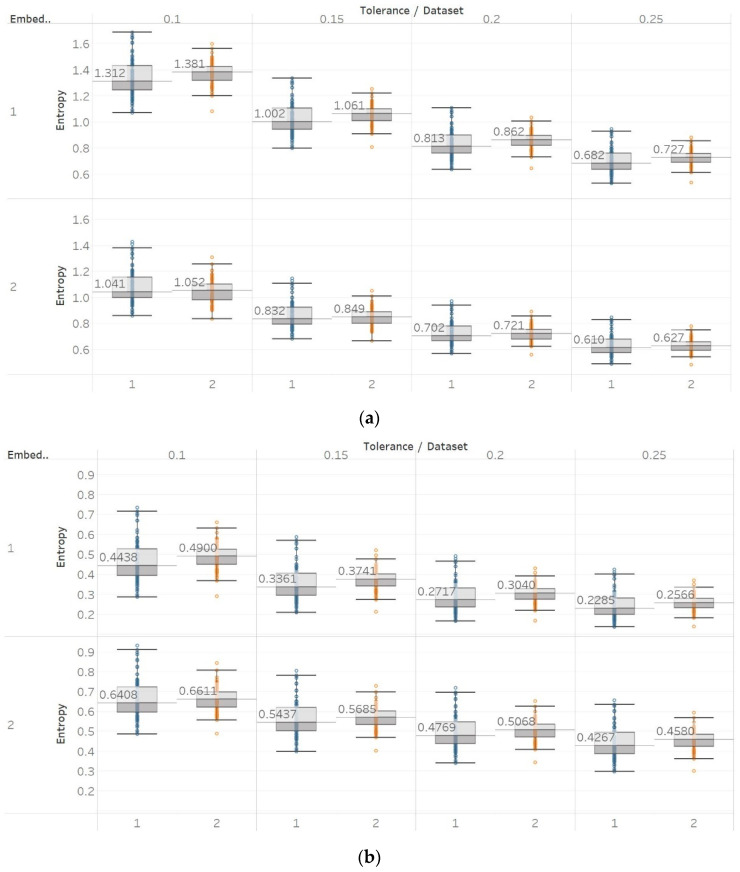

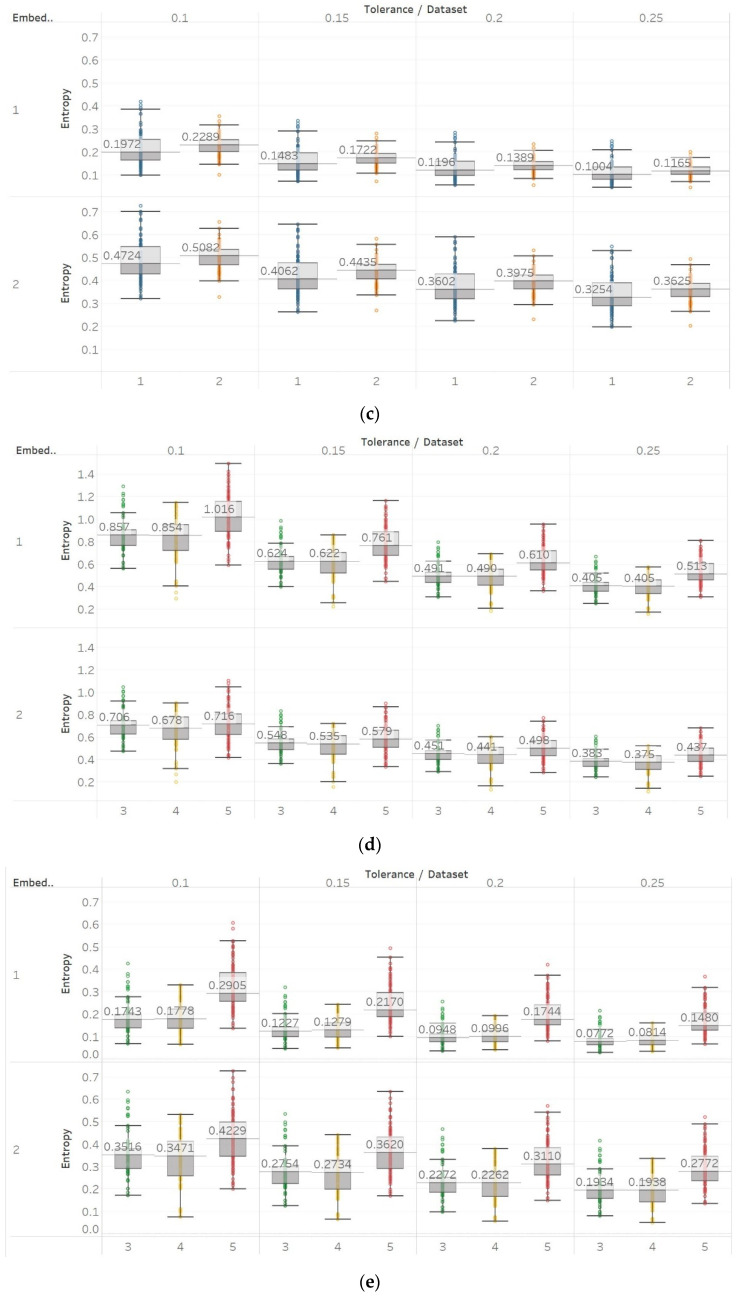

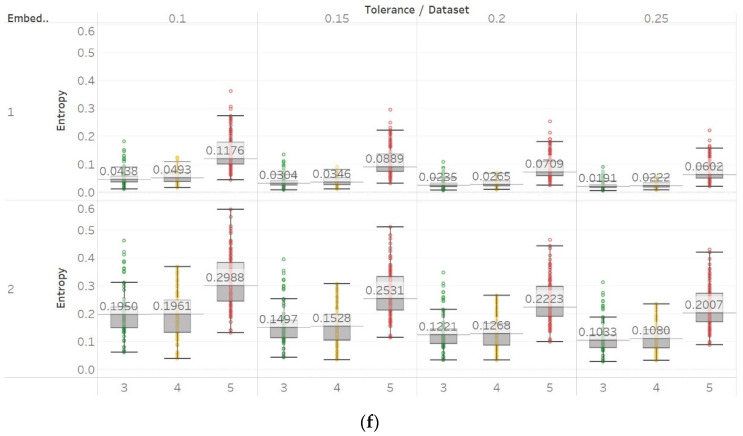
FEn results of electroencephalogram and epilepsy study (*I*): (**a**) FEn (n = 1) for group A; (**b**) FEn (*n* = 2) for group A; (**c**) FEn (*n* = 3) for group A; (**d**) FEn (*n* = 1) for group B; (**e**) FEn (*n* = 2) for group B; (**f**) FEn (*n* = 3) for group B. Data sets: (1) Eyes open healthy EEG; (2) eyes closed healthy EEG; (3) opposite hemisphere epileptic EEG; (4) epileptogenic zone epileptic EEG; (5) seizure EEG.

In FEn analysis, we tested 24 input parameter combinations created by using *m* = [1, 2], *n* = [1, 2, 3] and *r* = [0.1, 0.15, 0.2, 0.25] (Figure 11). Generally, results follow the same trend as for SEn and QSEn with *m* = 1, but are different to some presented in the literature [31]. This means that increased entropy is observed in closed-eyes EEG in group A and in seizure EEG segments in group B. These results suggest that FEn is sensitive to different signal dynamics compared to the previous algorithms while this can also vary depending on its input parameter combination. Nevertheless, FEn has previously shown increased absolute entropy values in the presence of a chaotic logistic map implementation (Figure 6), and therefore, inconsistencies in results here were not completely unexpected. It is worth noting though that using *n* = 1 and *m* = 2 produces results that are closer to the theoretical expectation, since FEn for the epileptogenic area data set is always lower than for the opposite hemisphere data set, while eyes-closed and seizure data sets have comparatively lower values than in other combinations. Finally, statistical analysis results suggest that observed differences are only significant (p≪0.01) for *m* = 1 and all *n, r* or for *n* = 3, *m* = 2, and *r* = [0.1, 0.15] in group A, while in group B we see significant differences between seizure and non-seizure groups for all parameter combinations except for *n* = 1, *m* = 2, and *r* = [0.1, 0.15] (Supplementary Material, Tables S1–S3). 

We also used k-NN to perform two separate classification tasks, one within each group (Supplementary Material, Tables S5 and S6). For group A, we have an average of 60% between all simulation accuracies while the maximum is 73.5% achieved by MPEn of *n* = 5. In general, PEn and MPEn achieve the highest scores (>64%) while the rest of the algorithms are significantly lower (<64.5%) and only occasionally manage to reach 60%. For group B, as expected, accuracies are generally lower because we have three classes instead of two. The average value is 48% while the maximum is 63% achieved by FEn of *n* = 3, *m* = 1, and *r* = 0.25. In general, SEn and QSEn give the lowest results in this task (<50%), PEn and MPEn stand in the middle (~50%), while FEn algorithms reach both low and high values (from 44% to 63%).

To improve the classification accuracy, we also trained the same k-NN algorithms and settings using two, three, four, and five features from different entropy algorithms combined as a feature vector (Supplementary Material, Table S7). We performed an analysis for all possible combinations of two, three, four, or five features to find the best results that are achievable under these settings. Group A accuracy ranged from 73.5% to 94%. Using two features (PEn of *n* = 5, FEn of *n* = 1, and *m* = 1, *r* = 0.2) increases the maximum accuracy from 73.5% to 92.5%, which then gradually saturates at 94% (MPEn of *n* = 5, SEn of *m* = 2, and *r* = 0.15; QSEn of *m* = 1 and *r* = 0.8; FEn of *n* = 3, *m* = 1, and *r* = 0.1). The group B maximum accuracy ranges from 63% to 87%. Using two features (SEn of *m* = 2 and *r* = 0.25; FEn of *n* = 2, *m* = 1, and *r* = 0.1) increases the accuracy from 63% to 83%, which then gradually saturates at 87% (PEn of *n* = 4; SEn of *m* = 2 and *r* = 0.25; FEn *n* = 3, *m* = 2, and *r* = 0.2).

### 3.3. Electroencephalogram and Epilepsy Studies (II)

**Figure 12:**
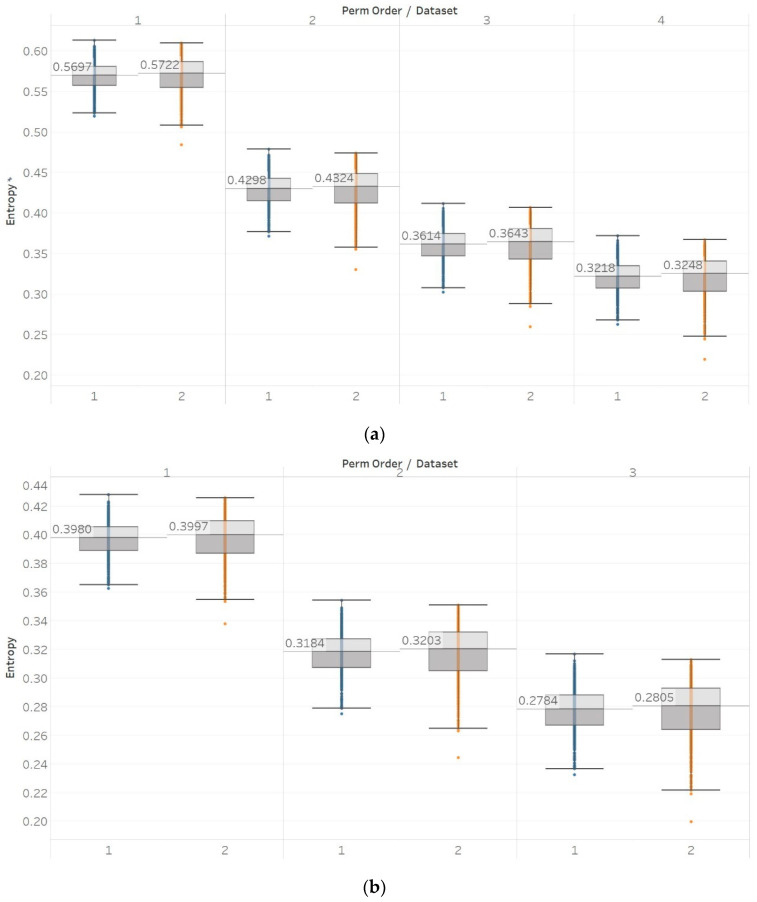
Electroencephalogram and epilepsy study (*II*) results: (**a**) PEn; (**b**) MPEn. Data sets: (1) Focal EEGs; (2) non-focal EEGs.

The same entropy algorithms were used to examine the differences between focal and non-focal EEG segments. Pen and MPEn both failed to discriminate between focal and non-focal channels almost completely (Figure 12). This is observed in the small differences between populations, as well as in the lack of statistically significant differences (*p* > 0.01 Supplementary Material, Table S4). However, it is worth noting that there is a slight tendency of non-focal entropy values to be higher, although this is mainly counterbalanced by the concurrent appearance of significantly lower values.

**Figure 13:**
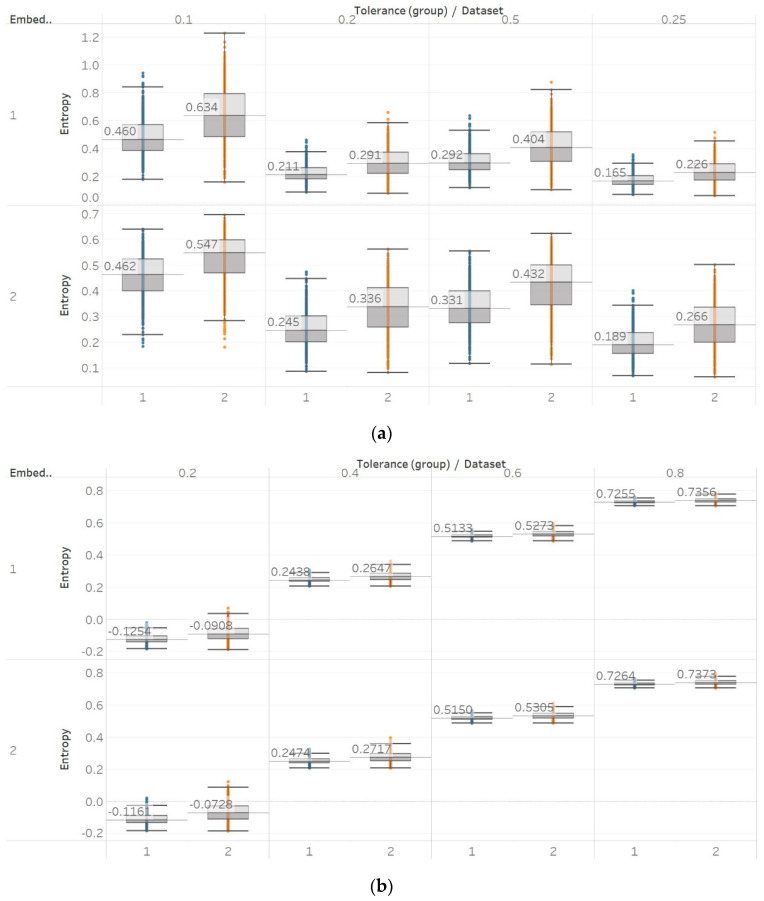
Electroencephalogram and epilepsy study (*II*) results: (**a**) SEn; (**b**) QSEn. Data sets: (1) Focal EEGs; (**2**) non-focal EEGs.

On the other hand, with Sen and QSEn and in contrast with the previous results, a substantial difference between focal and non-focal entropies was observed (Figure 13). This was also maintained across all different input parameter combinations. More specifically, non-focal EEG channels are characterised by increased Sen and QSEn values with results being statistically significant (*p* ≪ 0.01) for all parameter combinations (Supplementary Material, Table S4). This observation is in accordance with results from the first epileptic study where the entropy of the opposite hemisphere was lower compared to the epileptogenic area. In addition, it supports the general assumption of increased entropy in non-focal signals as the epileptic activity is described by more deterministic and defined signal structures compared to the non-focal increased randomness [30]. The only difference between the two algorithms is the increased presence of outliers in the latter.

**Figure 14:**
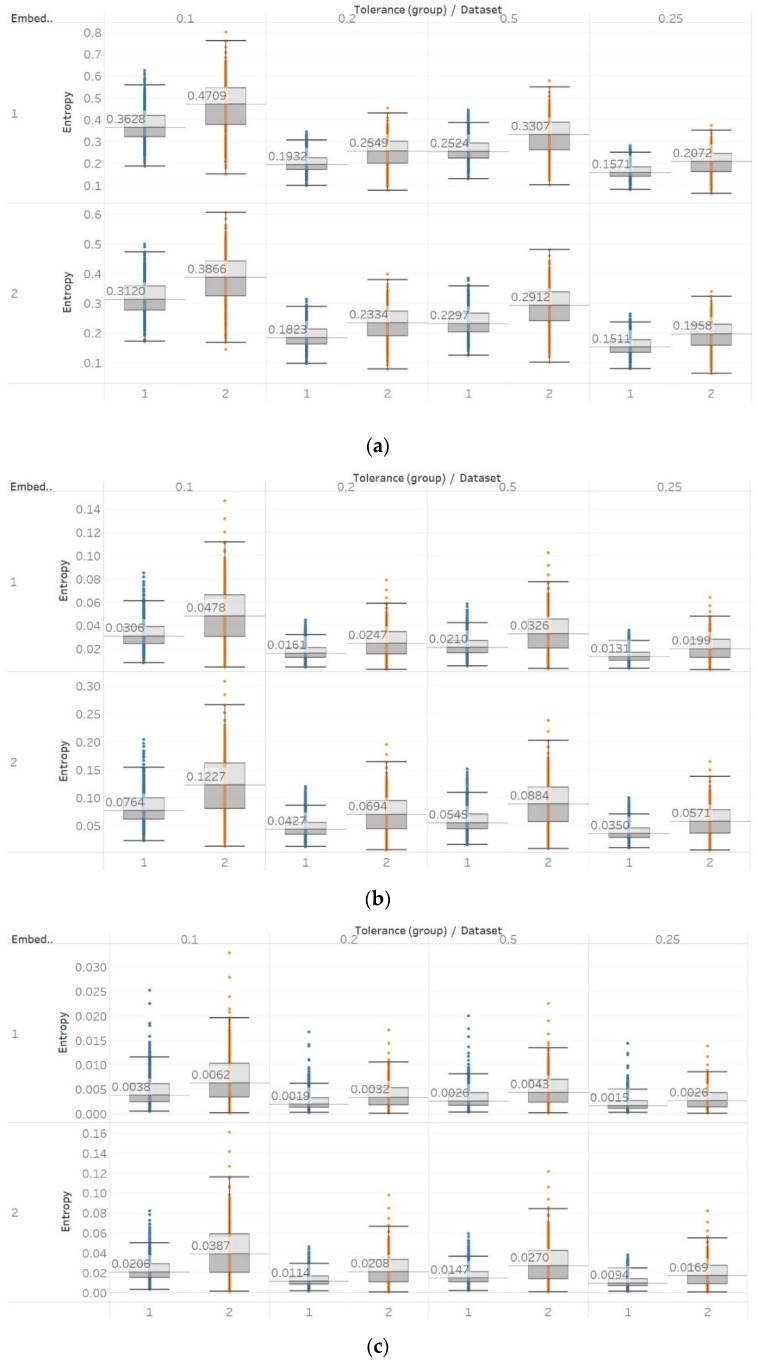
Electroencephalogram and epilepsy study (*II*) results: FEn: (**a**) *n* = 1; (**b**) *n* = 2; (**c**) *n* = 3. Data sets: (1) Focal EEGs; (2) non-focal EEGs.

Finally, FEn also produced similar results to those of SEn and QSEn, especially for *n* = 1 (Figure 14). The FEn of non-focal EEG segments is evidently higher compared to focal signals. This is also enhanced by the fact that while this difference is similar to those observed by the previous algorithms, the variances of populations appear to be considerably smaller. This would render FEn a better candidate for classification as there would be less overlap between the entropy values of the two data sets. Moreover, the differences between focal and non-focal *x* signals are statistically significant (p≪0.01) for all combinations of input parameters (Supplementary Material, Table S4). However, using *n* = 2 or *n* = 3 is not recommended, as both choices produce a lot of outliers while pushing the entropy values very close to zero as well.

To test whether the channel (focal versus non-focal) of an EEG segment can be predicted based on its entropy value, we also trained a classification algorithm. We used the same k-NN algorithm and methods as before for consistency. Results showed similar accuracies for most algorithms, with percentages ranging from 50% (obtained using FEn of *n* = 3, *m* = 1, and *r* = 0.2) to 63% (FEn of *n* = 1, *m* = 1, and *r* = 0.1). Those numbers are considered low, provided the fact that the algorithm is classifying only between two classes. Apart from the fact that the entropy values are similar for the two data sets, this outcome is further attributed to the insignificant differences in PEn-based algorithms and to the increased values of standard deviations observed previously in embedding entropy algorithms. However, when the number of features used is increased to two, we observe a maximum possible accuracy of 73% (using MPEn and SEn) which is then almost saturated for even larger feature vectors (75.5% with five features). A complete set of results is included in the Supplementary Material (Tables S8 and S9).

## 4. Discussion

In this study, the performance of five entropy algorithms in characterising biomedical signals was analysed. Before applying them to real signals, a thorough analysis of ten synthetic signals was carried out with each entropy algorithm. The signals were chosen in accordance with the relevant literature in order to have a common point of reference and to lay the ground for possible comparisons between non-linear analysis methods.

Through the synthetic signal analysis, we observed the main signal characteristics that affect the calculated entropy value of each algorithm. Interestingly, we observed almost complete consistency between the different algorithms, as all ten signals produced the same types and shapes of curves. In addition, the curves maintained their shapes for all variations of input parameters, and therefore, no absolute conclusion can be extracted on the optimal parameter values for any algorithm. In general, all five entropy algorithms are linearly affected by the increase of a single harmonic’s frequency, exponentially affected by the increase in the number of harmonics, linearly affected by WGN noise in high signal-to-noise ratio environments, and logarithmically affected by the increase in noise bandwidth during an AR process. In addition, they are all able to successfully detect a transition from randomness to orderliness in a MIX process, from periodicity to chaos in a logistic map and from chaos to periodicity in a Lorenz system. The highest entropy is observed when a signal is characterised by randomness, while entropy values for chaotic behaviour and periodicity can surpass each other depending on the level of regularity contained in their particular realisation. Permutation entropy algorithms (PEn and MPEn) are significantly faster to compute than entropies of the embedding family (SEn, QSEn, and FEn). The latter appear to be more accurate with noisy or complex signals with subtle transitions between different states. Although no absolute conclusion can be extracted for the optimal entropy algorithm based on the synthetic signals analysis alone, FEn seems to have increased sensitivity in signal changes while it also offers more possibilities for parameter fine-tuning. Nevertheless, all of the entropy algorithms but FEn had the same saturated response against a WGN signal, suggesting that FEn might be unique in being more affected by changes in the power of WGN (Figure 6). 

These observations are consistent with the relevant literature, in which similar synthetic signals were analysed using Lempel–Ziv complexity (LZC), ApEn, auto-mutual information rate of decrease (AMIRD) and SEn [28,32,33,34]. However, although LZC produced similar results to our study, it failed to detect changes in a signal with an increasing number of harmonics, unlike the entropy algorithms analysed herein. Moreover, the study with AMIRD provided identical results for the AR and MIX process signals, while it could only detect changes in a Lorenz system in the window that contained both chaotic and periodic behaviour, similar to results obtained using SEn of *m* = 2 (Figure 4). 

Through the application of entropy algorithms to real biomedical signals, we attempted to use their sensitivity to changes in signal complexity to distinguish between different classes of signals. EEG signals from two different epilepsy studies were used, due to the reported changes in signal complexity associated with epileptic activity [29,30]. In healthy subjects, we observed significant differences between surface EEG recordings that were made with eyes open or closed for all permutation entropy (PEn and MPEn) algorithm combinations and for most of the embedding entropy (SEn, QSEn, and FEn) algorithms. However, the former showed a decrease in entropy in closed-eyes EEG while the latter showed mostly the opposite. Permutation entropy algorithms are in line with the literature in which closed eyes are associated with the appearance of alpha brain rhythms, which are characterised by regularity [6]. On the contrary, though, another study that has compared the results of a Shannon-based entropy algorithm (namely Distribution Entropy) to FEn shows opposite results to what we have for both algorithm families [31]. Nevertheless, it is worth noting that distribution entropy is not the same as PEn or MPEn. Furthermore, the approach used in [31] is quite different to the one we have used in our study, as windows of different length were used to compute entropy (in our study the entropy of the whole signal was calculated instead), intracranial recordings from different groups in the data sets were combined to perform a comparison between ictal and interictal EEGs (we kept them separate in our study), and different types of EEGs were compared in the classification between the healthy (surface EEG) and epileptic (intracranial EEG) groups. In our study, for recordings made from the epileptogenic zone of the brain, the opposite hemisphere and during seizures, significant differences were mostly detected between the seizure and non-seizure recordings, while the differences between the brain zones during interictal periods were always significant for permutation entropy algorithms and never significant for embedding algorithms. At the same time, permutation entropy algorithms returned lower entropy values from the seizure EEG segments, followed by the epileptogenic zone, while embedding entropy algorithms returned mostly the opposite (apart from SEn of *m* = 2, Figure 9). For permutation entropy algorithms, results are in complete accordance with the literature [6,35,36]. In all these studies, PEn was used to either classify either between healthy and epileptic, or between ictal and interictal EEG segments and was found to decrease during ictal compared to interictal segments. On the other hand, results from SEn, QSEn, and FEn are less consistent across the literature. FEn analysis using the same data set has been found to produce higher entropy in seizure signals, followed by the epileptogenic zone and then by the opposite hemisphere signals [37]. SEn analysis from the same study has been found to produce higher entropy in the epileptogenic signals, followed by the seizure signals, and finally the ones from the opposite hemisphere. Additionally, studies that used other entropy or complexity measures, such as ApEn, LZC, and Spectral Entropy, have also reported increased values during seizures compared to interictal EEG segments [38,39]. All these are similar to what we have reported (Figure 9, Figure 10 and Figure 11). On the other hand, a different study [40] using the first EEG data set (i.e., the one originally published in [29]) reports increased SEn in ictal versus interictal signals. Nevertheless, there are several differences between our study and [40], as the latter focused on the analysis of shorter 5 s epochs extracted from the original EEG recordings, combined signals from the opposite hemisphere and the epileptogenic zone into a single interictal data set, and extended the set of input parameters used in the calculation of SEn beyond the ranges originally recommended by Richman and Moorman [10] and used in our study. Moreover, another study using the intracranial EEG data set from [29] and the whole signal length supports the claims of reduced SEn (calculated with *m* = 0.2 and *r* = 0.2) in recordings containing ictal activity when compared to epochs with interictal EEG activity [41].

To further investigate these observations, we used the second epilepsy data set, which included intracranial EEG recordings from epilepsy patients during interictal periods from focal and non-focal channels. In contrast with previous findings, permutation entropy algorithms in this case failed completely to detect any difference between focal and non-focal signals, while most embedding algorithms showed a statistically significant increase in entropy in non-focal signals (Figure 12, Figure 13 and Figure 14). This observation is also consistent with the literature where SEn and FEn have shown the same results on the same data set [42].

As a result, permutation entropy algorithms performed well in differentiating between signal classes in all tests except for the focal and non-focal signals. On the other hand, embedding algorithms performed well in all statistical tests except for the insignificant differences between epileptogenic and opposite hemisphere data sets, but their results in the first epilepsy database were inconsistent and dependent on input parameter combinations. Combining these conclusions with results from the literature, permutation entropy algorithms seem to have the advantage of significantly faster computation time and consistency, but they are not as sensitive (proven by failing to detect differences between focal and non-focal signals from the second data set). On the other hand, embedding entropy algorithms have increased sensitivity (proven by the differentiation between focal and non-focal signals) while also potentially having greater potential with correct tuning of their input parameters. However, this tuning is not trivial and might result in different results than expected. In terms of classification tasks, permutation entropy algorithms performed better in classifying data in the first data set (reached the maximum accuracy of 73.5%) while embedding algorithms performed better in the second data set (reached the maximum accuracy of 63%). Nonetheless, it is worth noting that using only one entropy algorithm as a feature generally yields low classification accuracies. These accuracies were equal to 73.5% for the classification between eyes-open and eyes-closed surface EEGs in healthy controls (obtained using PEn with *n* = 5); 63% for the classification between intracranial EEGs from epileptic patients recorded at the epileptogenic area, the opposite hemisphere and during seizures (obtained using FEn with *n* = 3, *m* = 1, and *r* = 0.2); and 63% for the classification between focal and non-focal intracranial EEGs (obtained using FEn with *n* = 1, *m* = 1, and *r* = 0.1). Increasing the number of features from one to five can increase the maximum possible accuracy significantly. This accuracy reached 94% for the first classification task using all algorithms except PEn (MPEn with *n* = 5; SEn with *m* = 2 and *r* = 0.15; QSEn with *m* = 1 and *r* = 0.8; FEn with *n* = 3, *m* = 1, and *r* = 0.1), 87% for the second classification task using all algorithms except MPEn (PEn with *n* = 4; SEn with *m* = 2 and *r* = 0.25; FEn with *n* = 3, *m* = 2, and *r* = 0.2), and 75.5% for the third classification task using all five entropy algorithms (PEn with *n* = 5; MPEn with *n* = 4; SEn with *m* = 2 and *r* = 0.15; QSEn with *m* = 2 and *r* = 0.25; FEn with *n* = 1, *m* = 1, and *r* = 0.15). It is worth noting than in all cases, the combination of results obtained with entropy algorithms from both families (i.e., based on Shannon’s entropy and based on embedding) clearly outperformed the results obtained by using methods from each family separately.

Our study has some limitations that should be highlighted. Firstly, although many synthetic signals were used to characterise the different entropy algorithms, there is scope to evaluate them with other synthetic data. This would provide additional and potentially valuable information about the way entropy algorithms characterise signals. In addition to that, the performance of other entropy algorithms (spectral entropy, wavelet entropy, etc.) could be analysed as well. In this way, we could find more common or uncommon responses between different entropy algorithms to synthetic and biomedical signals. Another limitation would be the classification techniques used and the extensiveness of the study as a whole. Our approach was limited to a pilot study, while trying to extend the theoretical analysis with a more practical approach and to provide a baseline for potential further studies. Finally, but equally importantly, the limited number of patients and EEG signals available and the limited number of signals used as part of the second epilepsy study, along with any potential bias of the data sets, should also be highlighted as a limitation.

## 5. Conclusions

Five different entropy algorithms (permutation, modified permutation, sample, quadratic sample and fuzzy entropy) were evaluated thoroughly using synthetic and biomedical signals. This should help interpret results obtained with these algorithms using different types of biomedical signals and facilitate comparisons between different studies. The synthetic signal analysis provided valuable insights on which signal characteristics are responsible for triggering a high or low entropy value for each of the entropy algorithms. All entropy algorithms proved to be highly sensitive to increased noise environments or randomness and moderately sensitive to chaotic or periodic behaviour. All algorithms were successful in differentiating between eyes-open and eyes-closed surface EEG signals from healthy controls, and between seizure and non-seizure intracranial EEG signals from patients with epilepsy. However, algorithms of the permutation family responded in an opposite way to those of the embedding family, with the former being closer to theoretical expectations. Consequently, more data sets are needed to confirm these findings about the possible usefulness of entropy algorithms in EEG analysis in epilepsy. Embedding entropies (sample, quadratic sample and fuzzy entropies) generally outperform the rest of the algorithms in terms of sensitivity and show greater potential by considering the fine-tuning possibilities they offer. On the other hand, permutation and modified permutation entropies are more consistent across different input parameter values and considerably faster to calculate. Finally, promising classification results were obtained using two (or more) different entropy measures as features, suggesting that complementary information can be obtained using different algorithms to improve the prediction of the class to which the EEG signals belonged to.

## Supplementary Materials

The following are available online at https://www.mdpi.com/1099-4300/21/9/840/s1, Table S1. Electroencephalogram and Epilepsy studies (I): *p*-values from Wilcoxon signed rank tests for group A. Tests that rejected the hypothesis at the *p* < 0.01 level are highlighted in bold, Table S2. Electroencephalogram and Epilepsy studies (I): *p*-values from the Kruskal–Wallis tests for group B. Tests that rejected the hypothesis at the *p* < 0.01 level are highlighted in bold, Table S3. Electroencephalogram and Epilepsy studies (I): Mann–Whitney U tests at *p* < 0.01 level for three pairs of data sets in group B: Data sets (3,4), data sets (3,5), data sets (4,5). Results of tests for the three pairs are given as *x*1, *x*2, and *x*3, where *xi* is equal to 1 if the hypothesis was rejected for that test and equal to 0 otherwise. Combinations that rejected the relevant Kruskal–Wallis test are underlined. Combinations that rejected all three Mann–Whitney U tests are highlighted in bold, Table S4. Electroencephalogram and Epilepsy studies (II): *p*-values from then Mann–Whitney U tests. Tests that rejected the hypothesis at the *p* < 0.01 level are highlighted in bold, Table S5. Electroencephalogram and Epilepsy studies (I): Classification accuracy results in group A using one of the entropy algorithms realisations as a feature and using *k* = 3. Maximum accuracy achieved is highlighted in bold, Table S6. Electroencephalogram and Epilepsy studies (I): Classification accuracy results in group B using one of the entropy algorithms realisations as a feature and using *k* = 3. Maximum accuracy achieved is highlighted in bold, Table S7. Electroencephalogram and Epilepsy studies (I): Best classification accuracy results in group A and group B for varying number of features used. Best in group accuracies are highlighted in bold, Table S8. Electroencephalogram and Epilepsy studies (II): Classification accuracy results using one of the entropy algorithms realisations as a feature and using *k* = 3. Maximum accuracy achieved is highlighted in bold, Table S9. Electroencephalogram and epilepsy studies (II): Best classification accuracy results for varying number of entropies used as a feature vector.
