# Supplementary material for: Interpretation of Entropy Algorithms in the Context of Biomedical Signal Analysis and Their Application to EEG Analysis in Epilepsy
Source: Entropy (Basel). 2019 Aug 27;21(9):840. doi: 10.3390/e21090840 (PMC7515369; doi:10.3390/e21090840)
Supplement: Supplementary file 1 [file entropy-21-00840-s001.pdf]

entropy

Supplementary Material

Interpretation of Entropy Algorithms in the Context of Biomedical Signal Analysis and Their Application to EEG Analysis in Epilepsy

Lampros Chrysovalantis Amarantidis [1,2] and Daniel Abásolo [1,*]

[1] Centre for Biomedical Engineering, Department of Mechanical Engineering Sciences, Faculty of Engineering and Physical Sciences, University of Surrey, Guildford, GU2 7XH, UK
[2] Ericsson, Thames Tower, Station Road, Reading, RG1 1LX, UK
* Correspondence: d.abasolo@surrey.ac.uk; Tel.: +44-(0)1483-682971

Received: 29 June 2019; Accepted: 23 August 2019; Published: date

Table S1. Electroencephalogram and Epilepsy studies (*I*): p – values from Wilcoxon signed rank tests for group A. Tests that rejected the hypothesis at the $p < 0.01$ level are highlighted in bold.

n	3	4	5	6				
PEn	**7.72e-14**	**4.11e-14**	**2.11e-14**	**1.90e-14**	-	-	-	-
MPEn	**7.72e-14**	**4.11e-14**	**2.11e-14**	-	-	-	-	-
(*m*,*r*)	(1, 0.1)	(1, 0.15)	(1, 0.2)	(1, 0.25)	(2, 0.1)	(2, 0.15)	(2, 0.2)	(2, 0.25)
SEn	0.0118	0.0120	0.0108	**0.0098**	**2.74e-10**	**5.92e-10**	**3.27e-09**	**4.85e-08**
(*m*,*r*)	(1, 0.2)	(1, 0.4)	(1, 0.6)	(1, 0.8)	(2, 0.2)	(2, 0.4)	(2, 0.6)	(2, 0.8)
QSEn	**0.0089**	**0.0077**	**0.0073**	**0.0057**	0.0230	0.2094	0.0131	**0.0037**
(*m*,*r*)	(1, 0.1)	(1, 0.15)	(1, 0.2)	(1, 0.25)	(2, 0.1)	(2, 0.15)	(2, 0.2)	(2, 0.25)
FEn, *n*=1	**0.0037**	**0.0035**	**0.0033**	**0.0036**	0.0900	0.4151	0.9233	0.6253
FEn, *n*=2	**0.0037**	**0.0037**	**0.0036**	**0.0036**	0.4555	0.1496	0.0643	0.0313
FEn, *n*=3	**0.0037**	**0.0038**	**0.0041**	**0.0040**	0.0313	0.0142	**0.0075**	**0.0048**

Table S2. Electroencephalogram and Epilepsy studies (*I*): p – values from the Kruskal-Wallis tests for group B. Tests that rejected the hypothesis at the $p < 0.01$ level are highlighted in bold.

n	3	4	5	6				
PEn	**1.46e-14**	**1.30e-14**	**7.07e-15**	**4.22e-15**	-	-	-	-
MPEn	**1.46e-14**	**1.30e-14**	**7.07e-15**	-	-	-	-	-
(*m*,*r*)	(1, 0.1)	(1, 0.15)	(1, 0.2)	(1, 0.25)	(2, 0.1)	(2, 0.15)	(2, 0.2)	(2, 0.25)
SEn	0.2363	0.6699	0.2932	0.0221	**1.73e-12**	**1.75e-11**	**3.54e-10**	**2.89e-08**
(*m*,*r*)	(1, 0.2)	(1, 0.4)	(1, 0.6)	(1, 0.8)	(2, 0.2)	(2, 0.4)	(2, 0.6)	(2, 0.8)
QSEn	**3.64e-07**	**6.16e-12**	**4.70e-15**	**4.29e-18**	0.0298	0.0787	**6.81e-08**	**1.44e-14**
(*m*,*r*)	(1, 0.1)	(1, 0.15)	(1, 0.2)	(1, 0.25)	(2, 0.1)	(2, 0.15)	(2, 0.2)	(2, 0.25)
FEn, *n*=1	**2.92e-12**	**3.13e-15**	**1.56e-17**	**3.58e-19**	0.1148	**0.0033**	**3.13e-05**	**2.88e-07**

FEn, *n*=2	**3.45e-27**	**3.99e-29**	**3.25e-30**	**6.52e-31**	**2.67e-08**	**9.97e-13**	**3.68e-16**	**6.82e-19**
FEn, *n*=3	**4.39e-34**	**5.26e-35**	**2.06e-35**	**1.11e-35**	**5.94e-19**	**5.43e-24**	**1.69e-26**	**3.19e-28**

Table S3. Electroencephalogram and Epilepsy studies (*I*): Mann-Whitney U tests at $p < 0.01$ level for three pairs of data sets in group B: data sets (3,4), data sets (3,5), data sets (4,5). Results of tests for the three pairs are given as x_1, x_2, x_3 where x_i is equal to 1 if the hypothesis was rejected for that test and equal to 0 otherwise. Combinations that rejected the relevant Kruskal-Wallis test are underlined. Combinations that rejected all three Mann-Whitney U tests are highlighted in bold.

n	3	4	5	6				
PEn	**1,1,1**	**1,1,1**	**1,1,1**	**1,1,1**	-	-	-	-
MPEn	**1,1,1**	**1,1,1**	**1,1,1**	**-**	-	-	-	-
(*m*,*r*)	(1, 0.1)	(1, 0.15)	(1, 0.2)	(1, 0.25)	(2, 0.1)	(2, 0.15)	(2, 0.2)	(2, 0.25)
SEn	0,0,0	0,0,0	0,0,0	0,0,0	0,1,1	0,1,1	0,1,1	0,1,1
(*m*,*r*)	(1, 0.2)	(1, 0.4)	(1, 0.6)	(1, 0.8)	(2, 0.2)	(2, 0.4)	(2, 0.6)	(2, 0.8)
QSEn	0,1,1	0,1,1	0,1,1	0,1,1	0,0,0	0,0,0	0,1,1	0,1,1
(*m*,*r*)	(1, 0.1)	(1, 0.15)	(1, 0.2)	(1, 0.25)	(2, 0.1)	(2, 0.15)	(2, 0.2)	(2, 0.25)
FEn, *n*=1	0,1,1	0,1,1	0,1,1	0,1,1	0,0,0	0,0,0	0,1,1	0,1,1
FEn, *n*=2	0,1,1	0,1,1	0,1,1	0,1,1	0,1,1	0,1,1	0,1,1	0,1,1
FEn, *n*=3	0,1,1	0,1,1	0,1,1	0,1,1	0,1,1	0,1,1	0,1,1	0,1,1

Table S4. Electroencephalogram and Epilepsy studies (*II*): p – values from then Mann-Whitney U tests. Tests that rejected the hypothesis at the $p < 0.01$ level are highlighted in bold.

n	3	4	5	6				
PEn	0.0641	0.0929	0.1476	0.2124	-	-	-	-
MPEn	0.0641	0.0929	0.1476	-	-	-	-	-
(*m*,*r*)	(1, 0.1)	(1, 0.15)	(1, 0.2)	(1, 0.25)	(2, 0.1)	(2, 0.15)	(2, 0.2)	(2, 0.25)
SEn	**5.75e-35**	**6.51e-35**	**1.27e-34**	**2.40e-34**	**5.90e-33**	**2.81e-34**	**9.31e-35**	**6.59e-35**
(*m*,*r*)	(1, 0.2)	(1, 0.4)	(1, 0.6)	(1, 0.8)	(2, 0.2)	(2, 0.4)	(2, 0.6)	(2, 0.8)
QSEn	**1.93e-33**	**4.29e-32**	**1.06e-30**	**2.94e-29**	**2.89e-34**	**1.00e-32**	**3.77e-31**	**1.37e-29**
(*m*,*r*)	(1, 0.1)	(1, 0.15)	(1, 0.2)	(1, 0.25)	(2, 0.1)	(2, 0.15)	(2, 0.2)	(2, 0.25)
FEn, *n*=1	**8.16e-37**	**2.20e-36**	**5.70e-36**	**9.36e-36**	**9.08e-36**	**4.08e-36**	**4.25e-36**	**4.26e-36**
FEn, *n*=2	**6.81e-30**	**1.93e-28**	**1.61e-27**	**7.95e-27**	**2.14e-35**	**1.85e-34**	**1.26e-33**	**6.22e-33**
FEn, *n*=3	**7.31e-17**	**2.76e-15**	**2.56e-14**	**1.17e-13**	**1.66e-29**	**2.68e-27**	**9.58e-26**	**1.79e-24**

Table S5. Electroencephalogram and Epilepsy studies (*I*): Classification accuracy results in group A using one of the entropy algorithms realisations as a feature and k-NN with *k*=3. Maximum accuracy achieved is highlighted in bold.

n	3	4	5	6				
PEn	70%	68.5%	73%	64%	-	-	-	-
MPEn	68%	69.5%	**73.5%**	-	-	-	-	-
(*m*,*r*)	(1, 0.1)	(1, 0.15)	(1, 0.2)	(1, 0.25)	(2, 0.1)	(2, 0.15)	(2, 0.2)	(2, 0.25)
SEn	56%	60%	64%	59%	57.5%	62%	61.5%	62.5%
(*m*,*r*)	(1, 0.2)	(1, 0.4)	(1, 0.6)	(1, 0.8)	(2, 0.2)	(2, 0.4)	(2, 0.6)	(2, 0.8)
QSEn	61.5%	58%	64%	69.5%	46%	57%	61.5%	61%
(*m*,*r*)	(1, 0.1)	(1, 0.15)	(1, 0.2)	(1, 0.25)	(2, 0.1)	(2, 0.15)	(2, 0.2)	(2, 0.25)
FEn, *n*=1	64.5%	59.5%	57%	60%	57.5%	52.5%	59%	55%
FEn, *n*=2	63.5%	63%	62.5%	56.5%	55.5%	58%	58%	66%
FEn, *n*=3	56%	53.5%	52.5%	53%	57.5%	63%	60%	57%

Table S6. Electroencephalogram and Epilepsy studies (*I*): Classification accuracy results in group B using one of the entropy algorithms realisations as a feature and k-NN with *k*=3. Maximum accuracy achieved is highlighted in bold.

n	3	4	5	6				
PEn	51%	50%	50%	49%	-	-	-	-
MPEn	48%	50%	48%	-	-	-	-	-
(*m*,*r*)	(1, 0.1)	(1, 0.15)	(1, 0.2)	(1, 0.25)	(2, 0.1)	(2, 0.15)	(2, 0.2)	(2, 0.25)
SEn	40%	41%	36%	37%	41%	41%	40%	50%
(*m*,*r*)	(1, 0.2)	(1, 0.4)	(1, 0.6)	(1, 0.8)	(2, 0.2)	(2, 0.4)	(2, 0.6)	(2, 0.8)
QSEn	43%	44%	50%	47%	36%	42%	45%	48%
(*m*,*r*)	(1, 0.1)	(1, 0.15)	(1, 0.2)	(1, 0.25)	(2, 0.1)	(2, 0.15)	(2, 0.2)	(2, 0.25)
FEn, *n*=1	46%	48%	51%	51%	46%	44%	44%	48%
FEn, *n*=2	48%	54%	52%	52%	50%	50%	50%	53%
FEn, *n*=3	58%	57%	62%	**63%**	46%	51%	55%	50%

Table S7. Electroencephalogram and Epilepsy studies (*I*): Best classification accuracy results in group A and group B for varying number of features used. Best in group accuracies are highlighted in bold.

1 feature	Features	Group A	Group B
Best in group A	**PEn** (*n*=5)	**73.5%**	48%
Best in group B	**FEn** (*n*=3, *m*=1, *r*=0.25)	53%	**63%**
2 features			
Best in group A	**PEn** (*n*=5), **FEn** (*n*=1, *m*=1, *r*=0.2)	**92.5%**	71%
Best in group B	**SEn** (*m*=2, *r*=0.25), **FEn** (*n*=2, *m*=1, *r*=0.1)	92.5%	**83%**
3 features			
Best in group A	**PEn** (*n*=3), **MPEn** (n=3), **FEn** (*n*=1, *m*=1, *r*=0.2)	**93.5%**	69%
Best in group B	**PEn** (*n*=4), **SEn** (*m*=2, *r*=0.25), **FEn** (*n*=3, *m*=2, *r*=0.15)	89%	**87%**
4 features			
Best in group A	**MPEn** (*n*=5), **SEn** (*m*=2, *r*=0.15), **QSEn** (*m*=2, *r*=0.25), **FEn** (*n*=3, *m*=1, *r*=0.1)	**94%**	81%
Best in group B	**PEn** (*n*=3), **SEn** (*m*=2, *r*=0.2), **QSEn** (*m*=2, *r*=0.25), **FEn** (*n*=3, *m*=2, *r*=0.15)	89%	**87%**

Table S8. Electroencephalogram and Epilepsy studies (*II*): Classification accuracy results using one of the entropy algorithms realisations as a feature and k-NN with *k*=3. Maximum accuracy achieved is highlighted in bold.

n	3	4	5	6				
PEn	50%	58.5%	52%	56%	-	-	-	-
MPEn	50.5%	59.5%	53.5%	-	-	-	-	-
(*m*,*r*)	(1, 0.1)	(1, 0.15)	(1, 0.2)	(1, 0.25)	(2, 0.1)	(2, 0.15)	(2, 0.2)	(2, 0.25)
SEn	59.5%	58%	56%	61%	52%	61.5%	57.5%	57%
(*m*,*r*)	(1, 0.2)	(1, 0.4)	(1, 0.6)	(1, 0.8)	(2, 0.2)	(2, 0.4)	(2, 0.6)	(2, 0.8)
QSEn	57%	60%	58%	54%	58%	55.5%	57.5%	56.5%
(*m*,*r*)	(1, 0.1)	(1, 0.15)	(1, 0.2)	(1, 0.25)	(2, 0.1)	(2, 0.15)	(2, 0.2)	(2, 0.25)
FEn, *n*=1	**63%**	62.5%	58%	59%	58.5%	58.5%	57%	57%
FEn, *n*=2	52.5%	55.5%	56.5%	57%	58.5%	59.5%	58%	55%
FEn, *n*=3	51%	51.5%	50%	55%	55%	58%	58.5%	55%

Table S9. Electroencephalogram and epilepsy studies (*II*): Best classification accuracy results for varying number of entropies used as a feature vector.

Number of features	Features	Best accuracy
1	**FEn** (*n*=1, *m*=2, *r*=0.15)	**63%**
2	**MPEn** (*n*=5), **SEn** (*m*=1, *r*=0.15)	**73%**
3	**MPEn** (*n*=3), **QSEn** (*m*=2, *r*=0.15), **FEn** (*n*=2, *m*=1, *r*=0.2)	**75%**
5	**PEn** (*n*=5), **MPEn** (*n*=4), **SEn** (*m*=2, *r*=0.15), **QSEn** (*m*=2, *r*=0.25), **FEn** (*n*=1, *m*=1, *r*=0.15)	**75.5%**

© 2019 by the authors. Submitted for possible open access publication under the terms and conditions of the Creative Commons Attribution (CC BY) license (http://creativecommons.org/licenses/by/4.0/).
